# Identifying fine motor difficulties in children with acute lymphoblastic leukemia: a scoping review

**DOI:** 10.1007/s00520-024-08667-0

**Published:** 2024-07-05

**Authors:** Silvia Hanna, Moatasem El-Ayadi, Faten Abdelazeim

**Affiliations:** 1 Department of Pediatric Physical Therapy, Faculty of Physical Therapy, Cairo University, Giza, Egypt; 2 Department of Pediatric Oncology, National Cancer Institute, Cairo University, Giza, Egypt; 3 Faculty of Physical Therapy, October 6th University, Giza, Egypt

**Keywords:** Leukemia, Motor skills, Weakness, Children

## Abstract

**Purpose:**

Survival rates for children diagnosed with acute lymphoblastic leukemia (ALL) have increased significantly over recent decades, and thus attention shifted toward understanding the adverse effects of cancer treatment. Chemotherapy has side effects that could affect muscle state and diminish motor performance. This scoping review was conducted to map the breadth of evidence for different tools used in fine motor skills assessment, the extent of upper extremity strength, and fine motor performance, highlighting the potential risk factors that may influence these skills.

**Methods:**

In March 2023, full-text studies that examined fine motor performance and/or upper extremity strength were identified via searches in PubMed, Science Direct, Scopus, Web of Science, and PEDro databases. The titles and abstracts of selected studies were screened according to the inclusion and exclusion criteria.

**Results:**

The search yielded initial 418 citations and 26 peer-reviewed articles were finally included in the review. Considerable heterogeneity was observed regarding the methods of evaluating fine motor skills. The results of this review indicate that children and adolescents with ALL experienced fine motor limitations and upper extremity weakness either during or after cessation of treatment.

**Conclusion:**

This scoping review presents a broad overview of the literature addressing fine motor difficulties in the pediatric population with ALL. Results accentuate the need to incorporate strengthening and occupational therapy training to preserve muscle strength and minimize future fine motor problems along the course of chemotherapeutic treatment. Little evidence was reported regarding the risk factors that may impair muscle strength and motor performance.

**Supplementary Information:**

The online version contains supplementary material available at 10.1007/s00520-024-08667-0.

## Introduction

Acute lymphoblastic leukemia (ALL) is the most common childhood cancer with an annual incidence rate of approximately 3.9 per 100,000 children, which accounts for over 75% of childhood leukemia [1, 2]. The highest incidence of ALL occurs during the first 5 years of life [3] that was more commonly observed in males as compared to females [4, 5].

Survival rates for childhood ALL have increased over the past five decades and reached about 90% [6]. Since the survival rate of leukemia has been improved, attention has shifted toward the understanding and treating of the adverse effects of cancer treatment [7].

Neurotoxicity from chemotherapy drugs affects both the central and peripheral nervous systems. Chemotherapeutic agents used in the management of childhood malignancies have side effects that can lead to a decrease in motor performance [8]. Treatment of ALL in children, mainly with vincristine and methotrexate, causes a long-standing axonal injury throughout the nervous system and demyelination within the spinal cord [9]. Motor and sensory-perceptual deficits can be considered important indicators of a compromised central nervous system. Chemotherapy induces oxidative stress in children with ALL, which in turn increases the risk of fine and visual-motor problems [10].

Generally, motor development is divided into fine and gross motor skills. Fine motor skills are necessary for developing basic self-help skills which involve using small muscles to manipulate objects [11, 12]. Fine motor functions have great biological importance for humans, as reflected by the large cortical representation of hands in the cerebral cortex [13]. During an ordinary school day, children spend between 30 and 60% of their school day performing fine motor tasks that involve the manipulation of writing implements, such as pencils, which are considered the most important skill in academic achievement [14]. Paper and pencil-based activities account for up to 85% of the time spent engaged in fine motor tasks [15].

Motor disability in children or survivors diagnosed with ALL is thought to be due to insufficient muscle activity leading to muscle weakness [16]. The resultant direct muscle fiber damage and hypoplasia adversely affect the function of the musculoskeletal system that subsequently lead to disuse and deconditioning with further weakness and impaired motor performance [8, 17].

Measures of fine motor control require manual dexterity and visual-motor integration [18]. Manual dexterity depends on prehension, coordination, and skills acquired through the practice of activities involving the manipulation of objects. Speed and precision are the commonly used criteria to measure this skill [19].

Few review studies discussed the effects of chemotherapeutic treatment on neuropsychological and neurocognitive functions that include fine motor skills or manual dexterity as a secondary aim [20–23]. The majority of these reviews focused on ALL survivors. In this review, we aimed to summarize clinical findings regarding the used evaluation tools, upper extremity strength status, and fine motor performance in children with ALL, as well as potential risk factors that may influence these skills.

## Methodology

### Search strategy

Studies published before December 2022 were searched on PubMed, Science Direct, Scopus, Web of Science, and PEDro databases using the keywords including (“acute lymphoblastic leukemia” OR “leukemia”) AND (“motor skills” OR “fine motor skills”) AND (“children” OR “childhood”) AND “strength” AND “chemotherapy”).

### Selection criteria

Papers meeting the following criteria were included: (1) studies considered children and adolescents diagnosed with ALL both during and/or after treatment; (2) articles assessed fine motor skills and/or upper extremity strength as a primary or a secondary aim, (3) full-text studies available online, and (4) articles published in English.

Intervention studies and articles that included mixed diagnoses and/or did not distinguish between fine and gross motor performance (had one global motor score) were excluded. We also excluded case reports, reviews, theses, or books. In addition, studies that used questionnaires to assess fine motor performance or muscular strength were not included in the current review. Selected studies were screened based on the title and abstract using the inclusion and exclusion criteria. Then, we reviewed the full text of potential studies and we also carefully reviewed the reference list of the included studies to find additional papers. In this review, we followed the Preferred Reporting Items for Systematic reviews and Meta-Analyses extension for Scoping Reviews (PRISMA-ScR) guidelines [24].

## Results

A total of 418 articles were identified initially through database searching, and then, they went down to 301 citations after duplicate removal (Supplementary file 1). Titles and abstracts were screened by two independent reviewers using the inclusion criteria, excluding 242 citations. Five additional articles were selected for inclusion through reference list review. The same reviewers then independently assessed the full text of 64 articles for inclusion according to the predefined eligibility criteria. Any disagreements between reviewers were resolved through a group discussion with an additional reviewer until the final consensus was reached.

**Fig. 1:**
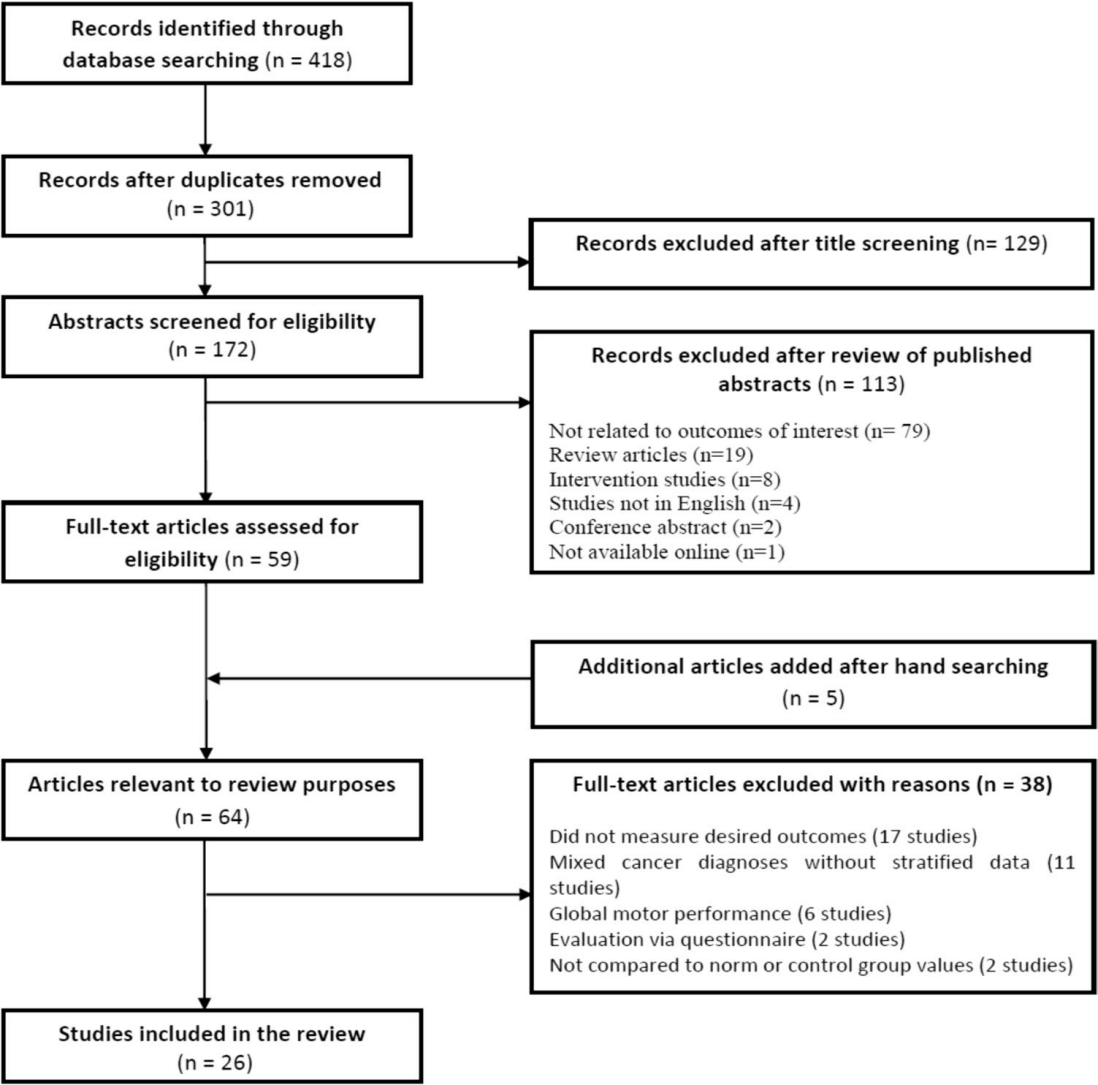
Flow diagram showing the selection process of studies

After reviewing the literature obtained during the search of the previous databases, a total of 26 studies were potentially eligible (Fig. 1). Since the timing of assessment would largely affect the results, we classified the selected papers according to whether they were conducted during or after the end of treatment for children with ALL. Twelve studies were applied to children receiving treatment, eleven studies were conducted after completion of therapy, and three studies included children on and off treatment. A detailed description of these studies is presented in Table 1.

**Table 1:** Characteristics and main findings of included studies

Study	Design	Time of assessment	Age at evaluation	Participants	Control group	Main domains assessed	Assessment tools of related domains discussed in this review	
							Fine motor function	Upper limb strength
I. Studies during treatment								
Dowell et al. [41]	Longitudinal	Within 3 months after diagnosis and again 1 year later	< 6 years of age at diagnosis (range = 33 to 66 months)	15 children with ALL (separated results) + 4 Burkitt’s lymphoma patients	Not applicable, results compared to age norms	- Cognitive skills - Gross motor functions - Fine motor functions - Visual-motor integration - Neurological functions	- The Grooved Pegboard - The Finger Tapping test	––-
Vainionpää [49]	Longitudinal	- On admission - After the induction therapy - 5–8 weeks after CNS therapy - 5 months after CNS therapy - 2–3 years after starting therapy	0.6–15.3y	40 (20 children received only chemotherapy and the other 20 participants received cranial irradiation)	Not applicable	Neurological examinations including the following: -Detailed tests of the cranial nerves -Tests of muscular tone and power -Deep tendon reflexes and cerebellar functions -Fine and gross motor movements	Tapping the finger tips against the thumb tip in a particular sequence	––-
Reinders-Messelink et al. [45]	Longitudinal	- Week 0 before 1st vincristine dose - 1 week after 1st vincristine dose - 1 week before 5th vincristine dose - 1 week after 8th vincristine dose - Week 46 (half year after 8th dose)	4–12 y	17 children with ALL	10 age- and sex-matched healthy children	- Gross and fine motor performance	Movement Assessment Battery for Children (Movement ABC)	––-
Hockenberry et al. [31]	Longitudinal	- Within 6 months of diagnosis - Annually for 2 years (year 1 and year 2, respectively during therapy)	3.1 to 14.6 years of age at the time of first evaluation	82 children with ALL	Not applicable, results compared to expected normative values	- Fine motor dexterity - Visual-motor integration - Intelligence	Purdue Pegboard	––-
Jansen et al. [32]	Cross-sectional	Within 2 weeks after start of the chemotherapy	4–13 y	50 children with ALL Only 29 performed fine motor assessment	29 healthy siblings Only 27 performed fine motor assessment	- Neuropsychological functioning - Intelligence	Purdue Pegboard	––-
Muratt et al. [28]	Cross-sectional	Children receiving maintenance therapy and undergone more than 6 months of treatment	12–16 y	Ten patients with ALL	Ten matched healthy children	- Isokinetic knee and elbow strength assessment	––-	A calibrated isokinetic dynamometer
Sabarre et al. [43]	Cross-sectional	During maintenance phase	5–16 y	15 children with ALL	Not applicable, results compared to standardized age-matched norms	- Manual dexterity	Movement Assessment Battery for Children– 2	––-
Ness et al. [25]	Cross-sectional	Outcomes were measured 7–10 days after diagnosis	4–18 y	109 children with ALL	Not applicable, results compared to age- and sex-specific expected values	- Bone mineral density - Body mass index - Ankle range of motion - Lower extremity strength - Grip strength - Motor development - Fitness - Health-related quality of life	––-	Jamar hand-held dynamometer
Hockenberry et al. [10]	Longitudinal	- Baseline evaluation, postinduction - Follow-up 1, during continuation, approximately one year from date of baseline evaluation - Follow-up 2, at the end of continuation therapy	2.9–15 y at the time of diagnosis	89 children with ALL	- Not applicable, results compared to national population norms	- Oxidative stress - Fine motor dexterity - Visual processing - Visual-motor integration - Behavioral adjustment	Purdue Pegboard	––-
Hanna et al. [54]	Cross-sectional	During maintenance phase	4–7 y	54 children with ALL	54 age and sex matched healthy peers	Fine motor functions (Fine motor precision, Fine motor integration, Manual Dexterity, Upper limb Coordination)	Bruininks-Oseretsky Test of Motor Proficiency second edition (BOT-2)	––-
Yildiz Kabak et al. [27]	Cross-sectional	During maintenance phase	5–15 y	30 children with ALL	Not applicable, results compared to normative values	- Pain - Fatigue - Grip strength - Motor proficiency - Fine motor functions - Functional capacity and physical performance of the lower limbs	- 9-Hole peg test - Grooved pegboard test	A digital dynamometer
Yildiz Kabak et al. [51]	Cross-sectional	During induction or consolidation chemotherapy	Mean age 9.88 y SD 2.33 y	25 children with ALL	21 age matched typically developing children	- Gross and fine motor functions - Cognitive performance	- 9-Hole peg test - Bruininks-Oseretsky Test of Motor Proficiency second edition- short form (BOT-2 SF)	––-
II. Studies after completion of treatment (ALL survivors)								
Reinders-Messelink et al. [46]	Cross-sectional	After completion of treatment at least 2 years	7–12 y	18 children with ALL	18 age- and sex-matched healthy children	- Fine motor skills - Handwriting skills	Movement Assessment Battery for Children–2	––-
Wright et al. [16]	Cross-sectional	> 12 months off therapy	5.5–14.5 y	36 survivors of ALL (Cranial irradiation was administered to 29 children)	36 age and gender matched control group	- Musculoskeletal and gross motor function - Hand grip strength	––-	A blood pressure cuff
Kingma et al. [34]	Longitudinal	The first assessment 3 to 6 months after cessation of chemotherapy then repeated at a median of 5 years later	- 4.2–8.10 y at 1st assessment - 7.1–12.11 y at 2nd assessment	17 survivors of ALL	225 healthy children of the same ages	- Verbal and performance intelligence - Auditory-verbal learning and memory - Sustained attention and speed - Visual-motor integration - Fine motor functioning	- Purdue Pegboard - Finger tapping test	––-
Kaemingk et al. [33]	Cross-sectional	3.90–11.68 years from diagnosis (i.e., 1.65–8.67 years from completion of treatment)	7.45–17.72 y	15 survivors of ALL	15 healthy control subjects Results also compared to normative values	- Math skills - Intellectual function - Basic reading skills - Verbal fluency - Visual-motor integration - Attention - Verbal and visual memory - Psychomotor speed	- Purdue Pegboard	––-
DeLuca et al. [44]	Cross-sectional	- Group 1: Less than 12 months post-treatment (0–12) - Group 2: 13 to 24 months post-treatment - Group 3: 25 to 60 months post-treatment	Total: 5–12 y (Group 1: 5–8 y; Group 2: 6.5–9.8 y; Group 3: 7–12 y)	A total of 37 ALL survivors (10 participants in group 1, 10 participants in group 2, and 17 participants in group 3)	- Not applicable, results compared to national population norms	- Gross and fine motor skills	Movement Assessment Battery for Children–2	––-
Balsamo et al. [40]	Cross-sectional	After completion of all therapy at least 1 year previously	6–17 y	256 of ALL survivors	- Not applicable	- Visual-motor integration - Fine motor functioning - Intelligence - Math calculation - Word reading	Lafayette Grooved Pegboard	––-
Van Der Plas et al. [39]	Cross-sectional	An average of 9.0 years (SD = 2.6) had passed since diagnosis	8–18 y	130 of ALL survivors	158 control subjects But motor speed and dexterity compared to normative values	- Working memory - Response inhibition - General cognitive abilities - Motor speed and dexterity - Math abilities - Executive functions - Parent-rated attention deficits and behavioral functions	Grooved Pegboard	––-
Goebel et al. [50]	Cross-sectional	Lag time since end of therapy from 0.3 to 14.0 y	6.1–18.3 y	31 ALL survivors	The tests were compared to three healthy age- and sex-matched control groups (i) 52 healthy subjects (presence of ataxia) (ii) 187 healthy subjects (testing fine motor function) (iii) 1142 healthy subjects (for testing postural isometric tremor)	- Fine motor function in terms of drawing and handwriting abilities - The presence and severity of cerebellar ataxia - Postural isometric tremor	A digitizing Tablet (DT)	––-
Lofstad et al. [38]	Cross-sectional	4.3–12.4 years post diagnosis	8.4–15.3 y	36 ALL survivors	36 healthy controls	- Neuropsychological functions including dexterity and grip strength as parts of motor function	- The Grooved Pegboard, - The Finger Tapping test	Not specified dynamometer
Oswald and Bo [37]	Cross-sectional	Currently receiving maintenance therapy or within two years of treatment cessation	5–18 y	13 (seven children completed treatment and six were receiving maintenance therapy)	13 healthy controls	- Motor functioning - Manual dexterity - Visual-motor integration - Academic skills	- Movement Assessment Battery for Children–2 - Grooved Pegboard	––-
van der Plas et al. [36]	Cross-sectional	Survivors had not received ALL treatment for a period of at least 2 years	8–18 y	71 ALL survivors	83 typically developing controls	- General cognitive abilities - Fine motor speed and dexterity - Behavioral function - Health-related quality of life	Grooved Pegboard Test	––-
III. On/off treatment studies								
Jansen et al. [30]	Longitudinal	- At diagnosis - 3 to 6 months after completion of treatment - 4.5 years after diagnosis	- 1st evaluation 4–11.8 y - 2nd evaluation 6.4–14.1 y - 3rd evaluation 8.2–16.3 y	- 1st evaluation 49 children with ALL - 43 children at 2nd evaluation - 40 children at 3rd evaluation	- 1st evaluation 29 healthy siblings - 27 healthy siblings at 2nd evaluation - 28 healthy siblings at 3rd evaluation	Neuropsychological assessments including measures of learning, memory, attention, speed, executive functioning, visual-constructive functioning, and fine-motor functioning	- Purdue Pegboard	––-
Akyay et al. [26]	Experimental prospective design	Two study groups: Group I were evaluated before the beginning of ALL chemotherapy (time 0) and at the end of the induction (time 1) In group II, measures were evaluated during their follow-up admissions (2–60 months after therapy)	4-year-old or more at the time of testing	- Group I included 15 newly diagnosed - Group II included 18 ALL survivors	Healthy matched age and sex control groups: 15 participants in control group I and 18 participants in group II	- Hand grip strength - Functional Mobility - Blood biochemistry - Muscle chemistry	––-	Pediatric Nicolas Hand-Held Dynamometer
Moore et al. [29]	Longitudinal	- Remission (baseline) - Annually for 3 years (years 1, 2, and 3)	- Age at baseline evaluation 3.1– 15.2 y - Age at year 1 evaluation 4.5– 16.3 y - Age at year 2 evaluation 5.5– 17.0 y - Age at year 3 evaluation 6.4– 17.9 y	71 ALL participants	Not applicable, results compared to age-adjusted standardized norms	- Cognitive assessments included measures of the following: * Fine motor dexterity * Visual-motor integration * Short-term verbal and visual memory - Academic abilities	- Purdue Pegboard	––-

### Assessment measures

Handgrip strength had been assessed using different types of dynamometers as Jamar, pediatric Nicolas, and digital hand-held dynamometers [25–27], in addition to using a blood pressure cuff [16]. Muratt et al. [28] measured the strength of elbow flexors and extensors by a calibrated isokinetic dynamometer.

With regard to the assessment of fine motor skills, the Purdue Pegboard Test (PPT) was the most frequently used measure to evaluate fine motor skills in previous literature [10, 29–34]. It can be used in the pediatric population as a brief, easy-to-administer test to assess fine motor dexterity and gross movement of the upper extremity by placing pegs into small holes with the dominant hand, non-dominant hand, and with both hands. The testing board consists of 4 cups at the top and two vertical rows, each row containing 25 small holes. It consists of four subtests and takes approximately 5 to 10 min to administer [35]. Another common measure used to assess motor speed and dexterity is the Grooved Pegboard Test (GPT) [27, 36–41]. The test can be used for children above 5 years of age and requires about 5 min to be completed by inserting pegs into 25 holes using the dominant hand, followed by the non-dominant hand. The score is recorded by the time required to place the pegs and calculated separately for each hand [42].

Five studies [37, 43–46] used the manual dexterity subtest of Movement Assessment Battery for Children–2 (MABC-2) to evaluate the fine motor performance. The MABC-2 is a standardized measure of motor functioning for children aged 3–16 years, including three tasks in the manual dexterity subtest [47].

Few studies used the finger-tapping test [34, 38, 41]. This test was designed to assess the fine motor speed and lateralized coordination using the index finger of the dominant and non-dominant hands [48]. Vainionpaa [49] simply examined the fine motor movements by tapping the fingertips against the thumb tip in a particular sequence, while Goebel et al. [50] assessed fine motor function only in terms of drawing and handwriting abilities using a Digitizing Tablet. Children were allowed to perform three tasks with the dominant hand. Also, the Nine-Hole Peg Test (9-HPT) was used in two studies conducted by Yildiz Kabak et al. [27, 51]. The participant is instructed to place and remove 9 pegs using the dominant hand then the non-dominant hand. The total time to complete the task was recorded in seconds that starts when the first peg is touched and ends when the last peg is dropped back in the container [52].

The Bruininks-Oseretsky Test of Motor Proficiency, Second Edition (BOT-2), is a standardized norm-referenced measure to measure global and fine motor proficiency in children aged from 4 to 21 years [53]. The fine motor form of BOT-2 was administered only in a single study on children with ALL [54]. It consists of the fine manual control and manual coordination composites that include fine motor precision (seven items), fine motor integration (eight items), manual dexterity (five items), and upper limb coordination subtests (seven items). Yildiz Kabak et al. [51] used the short form of the BOT-2 which consists of 14 items that can be used as a screening tool to achieve rapid and easy scoring, reflecting overall motor proficiency [53].

### Upper extremity weakness

Despite the limited number of available studies, distal upper extremity weakness has been recorded early in children with ALL, even within 7 days after diagnosis [25]. By the end of the acute treatment phase and before starting the maintenance phase, Akyay et al. [26] reported a significant reduction of grip strength in children with leukemia when compared to a healthy control group. This weakness tends to continue during the maintenance therapy as children had poor handgrip strength that was 60% of the norm-referenced values [27]. A previous study investigating isokinetic upper limb strength in children receiving maintenance therapy revealed that they had weaker elbow extensor strength than their healthy counterparts [28].

Regarding ALL survivors, Akyay et al. [26] and Lofstad et al. [38] reported that after cessation of treatment, grip strength was within normal range. On the contrary, Wright et al. [16] showed that the hand grip strength of the ALL survivors was significantly weaker than that of the comparison group. The difference in the results of this study may be attributed to the high proportion of survivors who received cranial irradiation (about 80% of the sample), which probably caused significant muscle weakness. Another possible factor was the difference in the hand grip evaluation tools used.

### Fine motor performance

#### During treatment

Shortly after diagnosis, no significant differences were found regarding fine motor skills between children with ALL and their siblings [32]. After starting the induction or consolidation phase, children with ALL started to develop fine motor impairments compared to typically developing children [51].

On maintenance therapy, Yildiz Kabak et al. [27] reported that the results of the GPT completion time did not differ from normative values and the 9-HPT completion time was 14% higher than the normative value. In contrast, Sabarre et al. [43] showed that more than 50% of children with ALL scored below average in fine motor skills, with 27% of children having significantly impaired scores. Also, Hanna et al. [54] reported that 67% of children undergoing maintenance therapy had fine motor difficulties in all domains of BOT-2, with 24% falling into the well-below-average category. Such variation of results during the maintenance phase may be attributed to inconsistency in assessment methods used in each study as well as differences in age groups included in these studies.

Because of the long duration of therapy that could reach up to 2 years, longitudinal studies in this period can reflect an in-depth picture of the existence of motor problems. Fine motor speed and dexterity for children had significantly less than the expected values at different phases of treatment [31]. Two longitudinal studies [10, 41] showed a significant improvement in fine motor skills over time that could reach the average range towards the end of therapy while only the performance of the dominant hand remained significantly below expectations [10].

On the contrary, another two longitudinal studies revealed that the incidence of fine motor impairments increased with time [45, 49]. Reinders-Messelink et al. [45] concluded that children with ALL, regardless of treatment phase or post–vincristine therapy, performed more poorly in fine motor skills when compared to a control group. After approximately 1 year of treatment, the percentage of fine motor problems increased to reach about 33%. According to Vainionpaa [49], no fine motor impairments have been detected at the time of diagnosis; however, fine motor impairments started to appear through the course of treatment. After 2–3 years of treatment, the percentage of fine motor impairments remained constant in intermediate and high-risk children receiving cranial irradiation, while the percentage of impairment increased to 33.3% in standard-risk children treated with chemotherapy only.

#### After completion of treatment

Most cross-sectional studies that investigated the fine motor skills after completion of therapy revealed that ALL survivors had poorer fine motor skills than their matched peers and/or normative values [33, 36–39, 46, 50]. However, only two studies reported that the fine motor functioning after completion of treatment was in the average range [40, 44]. This may be due to the lack of a control group in these studies, as the results of both studies were only compared to standard values. Regarding the longitudinal analysis, after cessation of chemotherapy, dexterity scores diminished over time and became below the average norm scores [34].

#### On/off treatment

Few studies have examined fine motor performance (2 studies) in children with ALL during and after completion of their course of treatment. No problems in neuropsychological outcomes have been detected at the time of diagnosis or after 3–6 months of completion of treatment, but children with ALL showed a significant decrease only in complex fine motor functions compared to healthy siblings 4.5 years after diagnosis [30]. Otherwise, Moore et al. [29] reported that fine motor dexterity improved over time but was significantly below the age-adjusted norms either at remission or after 3 years from the baseline evaluation.

### Factors that might affect fine motor performance

#### Cumulative chemotherapeutic doses

Along the course of treatment, no relationship was found between fine motor performance and cumulative doses of vincristine [43, 45, 54], methotrexate, or dexamethasone [54]. A longitudinal study conducted by Dowel et al. [41] revealed that the fine motor improvements significantly corresponded with the decrease in the number of vincristine and L-asparaginase administrations while they found no relation with respect to methotrexate. In addition, among ALL survivors, no relation was detected between fine motor functions and vincristine administration doses [46].

#### Gender effect

Two studies reported that girls had better manual dexterity scores than boys [30, 46], while only one study demonstrated a significant outperformance of boys in different fine motor domains [54]. Otherwise, most studies did not identify any significant difference in hand strength [16, 26] or fine motor skills in relation to gender in ALL survivors [37, 40, 44, 50]. The variation in results seems reasonable because these studies examined children with ALL at different ages and phases of treatment and most were after the end of treatment. There is only one study [54] that addressed the effect of gender on fine motor skills at an early age during treatment, while the remaining studies included children and adolescents of ALL survivors.

#### Age at diagnoses/assessment

Oswald and Bo [37] found that the older the age at diagnosis, the lower the performance on the Grooved Pegboard for both hands in ALL survivors. The results of this study should be interpreted with caution because the sample included a wide age range (5–18 years) of participants who were actively receiving maintenance treatment in addition to children who had completed their medical treatment. Goebel et al. [50] did not find any correlation between younger age and impaired motor performance; however, they found that older age at diagnosis was associated with higher variability on the more complex task of digitizing tablet. Other previous studies revealed no significant association between fine motor impairments and age at diagnosis [30, 40, 44] and/or age at assessment [44, 54].

#### Risk classification

Few studies addressed the effect of different ALL risk groups on muscle strength and fine motor skills. During the maintenance treatment, Hanna et al. [54] reported that the overall fine motor performance of children treated according to the standard-risk protocol was significantly better than that of the low-risk group. ALL risk groups did not affect manual dexterity along the course of treatment [31] or hand strength after completion of therapy [16].

## Discussion

Pediatric cancer treatment has negative sequelae that may impair levels of physical activity and fitness in pediatric cancer survivors compared to their healthy peers [55]. Alterations in motor performance, limitations in daily living activities, and muscle weakness were reported as the main priorities for physical rehabilitation of children and adolescents with cancer [56]. Incorporating the entire rehabilitation team and evaluating these priorities at the beginning, during, and after cancer treatment are beneficial to improve patient care by proactively identifying motor deficits. In addition, early and continuous rehabilitation allows for appropriate motor development as well as improvement of the child’s daily living activities and fine motor skills that may be affected by distal muscle weakness or atrophy [57].

In the current review, we aimed to discuss the state of upper extremity weakness in children diagnosed with ALL, as sufficient muscle strength is crucial to ensure successful fine and executive functions [58] and is directly correlated with functional motor tasks [59]. In this review, though there was no strong evidence that ALL survivors experienced muscle weakness after cessation of therapy, early muscle weakness in the upper extremities was reported along the course of treatment [25–28]. Therefore, these children may benefit from periodic evaluation of upper extremity strength and physical therapy intervention. Nielsen et al. [60] reported that children with ALL who underwent 3 months of supervised physical activity intervention maintained their baseline levels of handgrip strength. Inappropriate space for rehabilitation and lack of funding or resources are the most commonly identified barriers for physical rehabilitation [56].

Fine motor skills require integration of manual abilities, cognitive skills, spatial organization, and visual-motor coordination, and have been identified as an integral part of elementary school education [14]. Participation in play or school may be affected in children with ALL due to hospitalization and fatigue [43, 56]. Frequent absence from school and difficulties in fine motor activities can decrease school-based participation that may exhibit significant difficulties with academic achievement in ALL survivors [23]. Fine motor skills are not necessarily evaluated only in hospitals and clinics, but can also be addressed at school and the necessary modifications can be made in the classroom by consulting an occupational therapist.

Therefore, evaluation of fine motor abilities is a challenge and requires an aggregate measure rather than separate component skills [61, 62]. A considerable heterogeneity was observed regarding the methods of evaluating children’s fine motor skills that ranged from specific tests to assess motor speed/dexterity (e.g., Finger tapping task, Purdue Pegboard Test, Grooved Pegboard test) to comprehensive tests that measure more than one aspect of fine motor skills (e.g., BOT-2, MABC-2).

According to Knight and colleagues [63], tasks of a timed measure nature (Purdue Pegboard test or Finger tapping test) that assess fine motor coordination fail to control for the impact of psychomotor speed which has been affected in those children. Also, the BOT-2 brief form is a screening tool and may not be as accurate as the complete form to detect the mild impairments that may have an impact on the participation in physical activity [64]. In addition, its final score does not distinguish between gross and fine motor scores, although differences between them are important to consider in clinical assessments and designing rehabilitation interventions [65].

The development of central and peripheral nervous systems of those children may be subtly affected by chemotherapeutic agents (e.g., Vincristine; Methotrexate) which may be reflected in the form of reduced motor performance on functional measures [63]. Vincristine is commonly used as a part of standard treatment of childhood ALL treatment protocols [64]. Peripheral neuropathy is the most frequent side effect of vincristine toxicity that presents usually within the first 3 months of treatment. It causes paresthesia and pain in the hands and feet, followed by muscle weakness, particularly in wrist extensors and dorsiflexors [66]. Accordingly, the impaired fine motor performance may be related to the peripheral neuropathic effects of vincristine [23, 50].

Although most studies in this review confirmed that these children had fine motor impairments throughout the treatment period, we could not confirm the course of progression of these skills over time, as some longitudinal studies indicated their improvement, while others indicated their deterioration. As for post-treatment, the included studies assessed fine motor skills at various times ranging from a few months to several years off-treatment, making it difficult to confirm the extent to which these skills have deteriorated.

Some research gaps were identified in our scoping review. First, there could be some risk of bias in the results of this review due to the lack of a control group in several studies [10, 25, 27, 29, 31, 40, 41, 43, 44, 49]. Indeed, ten of the included studies did not have a control group, but they compared their results with normative values. Gaul and Issartel [67] claimed that during early school age children’s proficiency in fine motor skills did not progress at the rate expected by normative data. Accordingly, further studies with matched age- and sex-control groups are highly recommended.

Second, the articles included in this review encompassed a wide range of different assessment measures that precluded the interpretation and generalization of results. Third, the relatively broad age range of the included studies including children and adolescents added further to the complexity of interpretation and analysis. Another possible limitation is the great variation of the evaluation time across reviewed studies, either during or after treatment. In addition, language bias might exist as the review was restricted to articles published in English. Moreover, one study in this review included ALL patients/survivors who were either actively receiving maintenance therapy or off-medical treatment.

## Recommendations

The findings from this scoping review highlight some important areas for future research in children diagnosed with ALL. Regular evaluation of fine motor performance along the course of chemotherapy could identify early impairments. The multidisciplinary cancer team, including physical and occupational therapists, has a crucial role in detecting and providing the optimal intervention program. Upper extremity strengthening and occupational therapy training are prompted to preserve muscle strength and to minimize future fine motor problems along the course of chemotherapeutic treatment. More work is needed to identify comprehensive, clinically relevant measurement tools that can truly reflect a real picture of all aspects of fine motor performance, including school-related fine motor tasks where children spend a large portion of their time. Evaluation of neuropathy induced by chemotherapeutic agents in children and adolescents with ALL during and after completion of treatment needs to be addressed in upcoming studies. In addition to the main potential risk factors such as age, gender, and risk classification, some other external factors may negatively impact the development of fine motor skills, such as the duration of hospitalization, the effect of fatigue, and time away from school. Consequently, addressing the causal relationship between the potential risk factors that may influence fine motor performance in future studies is recommended.

## Conclusion

In conclusion, this study provides an updated summary of fine motor difficulties in the pediatric population with ALL. Results from this review indicate that those children experienced fine motor limitations and upper extremity weakness either during or after cessation of treatment. However, there is little evidence regarding the risk factors that may impair muscle strength and motor performance.

Overall, early physical rehabilitation is recommended once these children are admitted in an attempt to minimize the complications of therapeutic agents.

### Supplementary Information

Below is the link to the electronic supplementary material.

Supplementary file1 (DOCX 47 KB)

## Data Availability

The data supporting the findings of this study are available within the article or its supplementary information.
