# Supplementary material for: Identifying fine motor difficulties in children with acute lymphoblastic leukemia: a scoping review
Source: Support Care Cancer. 2024 Jul 5;32(7):488. doi: 10.1007/s00520-024-08667-0 (PMC11226483; doi:10.1007/s00520-024-08667-0)
Supplement: Supplementary file 1 — Supplementary file1 (DOCX 47 KB) [file 520_2024_8667_MOESM1_ESM.docx]

**Identifying Fine Motor Difficulties in Children with Acute Lymphoblastic Leukemia: A Scoping Review**

**Journal:** Supportive Care in Cancer.

**Authors**: Silvia Hanna, PhD;^1^ Moatasem El-Ayadi, MD;^2^ Faten Abdelazeim, PhD^1,3^

**Affilations:**

^1^Department of Pediatric Physical Therapy, Faculty of Physical Therapy, Cairo University, Egypt.

^2^Department of Pediatric Oncology, National Cancer Institute, Cairo University, Egypt.

^3^Faculty of Physical Therapy, October 6^th^ University, Cairo, Egypt.

| **Corresponding Author** |  |
| --- | --- |
| **Name** | Silvia Hanna |
| **Address** | 7 Ahmed Elzaiat St. Ben Elsaryat, El Dokki, Giza, Egypt |
| **Phone** | **+002** 01270281053 |
| **Email** | [silvia.hanna@pt.cu.edu.eg](mailto:silvia.hanna@pt.cu.edu.eg) |

**Supplementary Table 1 – Articles identified after removal of duplication**

| **N** | **All identified 301 records** | **Data bases** |
| --- | --- | --- |
|  | Motor skill delays in pre-school children with leukemia one year after treatment: Hematopoietic stem cell transplantation therapy as an important risk factor | **Scopus** |
|  | Investigation of the effect of task-orientated rehabilitation program on motor skills of children with childhood cancer: A randomized-controlled trial | **Scopus** |
|  | **Gross and fine motor skills in children treated for acute lymphoblastic leukaemia** | **Scopus** |
|  | Oncological children and well-being: Occupational performance and hrqol change after fine motor skills stimulation activities | **Scopus** |
|  | **The association between motor skills and academic achievement among pediatric survivors of acute lymphoblastic leukemia** | **Scopus** |
|  | **Longitudinal evaluation of fine motor skills in children with leukemia** | **Scopus** |
|  | **Motor functioning and associated cognitive outcomes in pediatric survivors of acute lymphoblastic leukemia** | **Scopus** |
|  | **Fine motor and handwriting problems after treatment for childhood acute lymphoblastic leukemia** | **Scopus** |
|  | **Investigating fine motor deficits during maintenance therapy in children with acute lymphoblastic leukemia** | **Scopus** |
|  | **Vincristine and fine motor function of children with acute lymphoblastic leukemia** | **Scopus** |
|  | Retrospective study of intellectual development in children treated for acute lymphoblastic leukaemia | **Scopus** |
|  | **Cognitive and behavioral risk factors for low quality of life in survivors of childhood acute lymphoblastic leukemia** | **Scopus** |
|  | Language and Motor Skills Are Impaired in Infants with Biliary Atresia Before Transplantation | **Scopus** |
|  | **Long-term gross motor performance following treatment for acute lymphoblastic leukemia** | **Scopus** |
|  | Neuromuscular mechanisms that contribute to gross motor performance in survivors of childhood acute lymphoblastic leukemia | **Scopus** |
|  | Does daily physical activity level determine the physical efficiency of children after treatment of leukemia? | **Scopus** |
|  | **Neuropsychological outcome in chemotherapy-only-treated children with acute lymphoblastic leukemia** | **Scopus** |
|  | Motor and Basic Cognitive Functions in Children with Acute Lymphoblastic Leukemia Undergoing Induction or Consolidation Chemotherapy | **Scopus** |
|  | Neurodevelopmental functioning of infants with untreated single-suture craniosynostosis during early infancy | **Scopus** |
|  | **Motor performance of children during treatment for acute lymphoblastic leukemia** | **Scopus** |
|  | A meta-analysis of the neuropsychological sequelae of chemotherapy-only treatment for pediatric acute lymphoblastic leukemia | **Scopus** |
|  | Developmental assessment of infants with biliary atresia: Differences between boys and girls | **Scopus** |
|  | Neuropsychological late effects of treatment for acute leukemia in children with Down syndrome | **Scopus** |
|  | Motor and perceptual timing deficits among survivors of childhood leukemia | **Scopus** |
|  | Effects of Central-Nervous-System Irradiation on Neuropsychologic Functioning of Children with Acute Lymphocytic Leukemia | **Scopus** |
|  | **Motor function in survivors of pediatric acute lymphoblastic leukemia treated with chemotherapy-only** | **Scopus** |
|  | Slight impairment of psychomotor skills in children after treatment of acute lymphoblastic leukemia | **Scopus** |
|  | **Analysis of handwriting of children during treatment for acute lymphoblastic leukemia** | **Scopus** |
|  | **Peripheral neuropathy in survivors of childhood acute lymphoblastic leukemia** | **Scopus** |
|  | Nerve lesions after therapy for childhood acute lymphoblastic leukemia | **Scopus** |
|  | Cognitive function in children with leukemia. Effect of radiation dose and time since irradiation | **Scopus** |
|  | A randomized trial investigating an exercise program to prevent reduction of bone mineral density and impairment of motor performance during treatment for childhood acute lymphoblastic leukemia | **Scopus** |
|  | **Feasibility and initial effectiveness of home exercise during maintenance therapy for childhood acute lymphoblastic leukemia** | **Scopus** |
|  | **Skeletal, neuromuscular and fitness impairments among children with newly diagnosed acute lymphoblastic leukemia** | **Scopus** |
|  | Improving body function and minimizing activity limitations in pediatric leukemia survivors: The lasting impact of the Stoplight Program | **Scopus** |
|  | Neuropsychologic assessment of long-term survivors of childhood leukemia | **Scopus** |
|  | **Associating physical activity levels with motor performance and physical function in childhood survivors of acute lymphoblastic leukemia** | **Scopus** |
|  | Nusinersen versus sham control in later-onset spinal muscular atrophy | **Scopus** |
|  | **Oxidative stress, motor abilities, and behavioral adjustment in children treated for acute lymphoblastic leukemia** | **Scopus** |
|  | Characterizing neurocognitive late effects in childhood leukemia survivors using a combination of neuropsychological and cognitive neuroscience measures | **Scopus** |
|  | **Neuropsychological effects of chemotherapeutic agents** | **Scopus** |
|  | Neurocognitive and neuroanatomical changes in children with acute lymphoblastic leukemia treated with the modified BFM-95 protocol | **Scopus** |
|  | The contribution of neurocognitive situation, physical capacity and daily life activities to quality of life in childhood acute lymphoblastic leukemia survivors | **Scopus** |
|  | Development of children born to mothers with cancer during pregnancy: Comparing in utero chemotherapy-exposed children with nonexposed controls | **Scopus** |
|  | Visuomotor function in children treated for acute lymphoblastic leukaemia with chemotherapy only | **Scopus** |
|  | Cognitive, educational, psychosocial adjustment and quality of life of children who survive hematopoietic SCT and their siblings | **Scopus** |
|  | Oxidative stress and executive function in children receiving chemotherapy for acute lymphoblastic leukemia | **Scopus** |
|  | Factors related to changes in cognitive, educational and visual motor integration in children who undergo hematopoietic stem cell transplant | **Scopus** |
|  | Assessment of Fundamental Movement Skills in Childhood Cancer Patients | **Scopus** |
|  | **Motor performance in children and adolescents with cancer at the end of acute treatment phase** | **Scopus** |
|  | **An evaluation of participation restrictions and associated factors via the ICF-CY framework in children with acute lymphoblastic leukemia receiving maintenance chemotherapy** | **Scopus** |
|  | Neuropsychologic effects of chemotherapy on children with cancer: A longitudinal study | **Scopus** |
|  | Chemotherapy-induced peripheral neuropathy in long-term survivors of childhood cancer clinical, neurophysiological, functional, and patient-reported outcomes | **Scopus** |
|  | Neurocognitive outcome and compensating possibilities in children and adolescents treated for acute lymphoblastic leukemia with chemotherapy only | **Scopus** |
|  | Neurocognitive development of children 4 years after critical illness and treatment with tight glucose control: A randomized controlled trial | **Scopus** |
|  | The cognitive effects of chemotherapy in post-menopausal breast cancer patients: A controlled longitudinal study | **Scopus** |
|  | Motor control in children with ADHD and non-affected siblings: Deficits most pronounced using the left hand | **Scopus** |
|  | Neurocognitive development of children after a cerebellar tumor in infancy: A longitudinal study | **Scopus** |
|  | Chemotherapy-related changes in central nervous system phospholipids and neurocognitive function in childhood acute lymphoblastic leukemia | **Scopus** |
|  | Cognitive and psychological outcomes of pediatric allogeneic hematopoietic stem cell transplantation survivors in a single center in China | **Scopus** |
|  | Are motor inhibition and cognitive flexibility dead ends in ADHD? | **Scopus** |
|  | **Neuropsychological test performance of pediatric cancer patients at diagnosis and one year later** | **Scopus** |
|  | Active video games to promote physical activity in children with cancer: A randomized clinical trial with follow-up | **Scopus** |
|  | Resistance training enhances muscular performance in patients with anorexia nervosa: A randomized controlled trial | **Scopus** |
|  | Differences in activities of daily living performance between long-term pediatric sarcoma survivors and a matched comparison group on standardized testing | **Scopus** |
|  | Close interrelation of motor development and cognitive development and of the cerebellum and prefrontal cortex | **Scopus** |
|  | Saliva collection technique for cytologic, microbiologic and viral evaluation in pediatric HIV infection | **Scopus** |
|  | Homeobox gene involvement in normal hematopoiesis and in the pathogenesis of childhood Leukemias | **Scopus** |
|  | Profound destructive effects of adolescent exposure to Vincristine accompanied with some sex differences in motor and memory performance | **Scopus** |
|  | Executive dysfunction 25 years after treatment with cranial radiotherapy for pediatric lymphoid malignancies | **Scopus** |
|  | Progressive declines in neurocognitive function among survivors of hematopoietic stem cell transplantation for pediatric hematologic malignancies | **Scopus** |
|  | Neuropsychological effects of childhood cancer treatment | **Scopus** |
|  | Visuomotor control in survivors of childhood acute lymphoblastic leukemia treated with chemotherapy only | **Scopus** |
|  | Linguistic-cognitive outcomes in children with acute lymphoid leukemia: An exploratory study | **Scopus** |
|  | What about School? Educational Challenges for Children and Adolescents with Cancer | **Scopus** |
|  | Impairment in circadian activity rhythms occurs during dexamethasone therapy in children with leukemia | **Scopus** |
|  | Central nervous system prophylactic treatment for childhood leukemia: Neuropsychological outcome studies | **Scopus** |
|  | **Neurocognitive predictors of academic outcomes among childhood leukemia survivors** | **Scopus** |
|  | Neuropsychological functioning of children treated for acute lymphoblastic leukemia: Impact of whole brain radiation therapy | **Scopus** |
|  | Neuropsychological effects of cranial irradiation in young children with acute lymphoblastic leukemia 9 months after diagnosis | **Scopus** |
|  | Increase in oxidative stress as measured by cerebrospinal fluid lipid peroxidation during treatment for childhood acute lymphoblastic leukemia | **Scopus** |
|  | Progressive neurocognitive impairment in young adult survivors of childhood acute lymphoblastic leukemia | **Scopus** |
|  | Changes in Oxidant Defense, Apoptosis, and Cognitive Abilities During Treatment for Childhood Leukemia | **Scopus** |
|  | Post-transplant adaptive function in childhood cerebral adrenoleukodystrophy | **Scopus** |
|  | **Vincristine-induced peripheral neuropathy in survivors of childhood acute lymphoblastic leukaemia** | **Scopus** |
|  | Attentional Deficits in Long-term Survivors of Childhood Acute Lymphoblastic Leukemia (ALL) | **Scopus** |
|  | Cerebellum development during childhood and adolescence: A longitudinal morphometric MRI study | **Scopus** |
|  | Predictive factors of internalized and externalized behavioral problems in children treated for acute lymphoblastic leukemia | **Scopus** |
|  | Tuberculosis-Associated HLH in an 8-Month-Old Infant: A Case Report and Review | **Scopus** |
|  | Voxel-based analysis of T2 hyperintensities in white matter during treatment of childhood leukemia | **Scopus** |
|  | Outcome of children with high-risk acute myeloid leukemia given autologous or allogeneic hematopoietic cell transplantation in the aieop AML-2002/01 study. | **Scopus** |
|  | F2-isoprostanes: A measure of oxidative stress in children receiving treatment for leukemia | **Scopus** |
|  | Visual Attention and Math Performance in Survivors of Childhood Acute Lymphoblastic Leukemia | **Scopus** |
|  | Psychological wellbeing in adolescents with leukaemia: A comparative study with typical development peers | **Scopus** |
|  | Motion Analysis of a Jumping Task in Childhood Leukemia Survivors | **Scopus** |
|  | Balance in children with acute lymphoblastic leukemia | **Scopus** |
|  | Neurocognitive disfunction in children with acute lymphoid leukemia [Disfunción neurocognitiva en niños con leucemia linfoide aguda] | **Scopus** |
|  | Longitudinal study of the neurodevelopmental characteristics of treated and untreated nonsyndromic craniosynostosis in infancy | **Scopus** |
|  | Perception of effort at low and moderate intensity exercise in survivors of childhood acute lymphoblastic leukaemia | **Scopus** |
|  | National, clinical cohort study of late effects among survivors of acute lymphoblastic leukaemia: The ALL-STAR study protocol | **Scopus** |
|  | Psychological development of the child with leukemia: A review | **Scopus** |
|  | Hematopoietic stem cell transplantation (HSCT) in children with juvenile myelomonocytic leukemia (JMML): Results of the EWOG-MDS/EBMT trial | **Scopus** |
|  | Perforin- and granulysin-mediated cytotoxicity and interleukin 15 play roles in neurocognitive impairment in patients with acute lymphoblastic leukaemia | **Scopus** |
|  | Neuropsychological assessment of attention in children with spina bifida | **Scopus** |
|  | Neuroimaging and neuropsychological follow-up study in a pediatric brain tumor patient treated with surgery and radiation | **Scopus** |
|  | Total-body irradiation and melphalan is a safe and effective conditioning regimen for autologous bone marrow transplantation in children with acute myeloid leukemia in first remission | **Scopus** |
|  | The developmental pathways of preschool children with acute lymphoblastic leukemia: Communicative and social sequelae one year after treatment | **Scopus** |
|  | Dexamethasone alters sleep and fatigue in pediatric patients with acute lymphoblastic leukemia | **Scopus** |
|  | Allogeneic bone marrow transplantation for chronic myelomonocytic leukemia in childhood: A report from the European Working Group on Myelodysplastic Syndrome in Childhood | **Scopus** |
|  | Assessment of brain changes with registered MR before and after bone marrow transplantation for chronic myeloid leukemia | **Scopus** |
|  | Neuropsychological differences between survivors of supratentorial and infratentorial brain tumours | **Scopus** |
|  | Short-Term Consequences of Pediatric Anti-cancer Treatment Regarding Blood Pressure, Motor Performance, Physical Activity and Reintegration Into Sports Structures | **Scopus** |
|  | Mathematics intervention for prevention of neurocognitive deficits in childhood leukemia | **Scopus** |
|  | Testing physical function in children undergoing intense cancer treatment—a RESPECT feasibility study | **Scopus** |
|  | Computerized cognitive training for amelioration of cognitive late effects among childhood cancer survivors: A randomized controlled trial | **Scopus** |
|  | Functional neurological outcome in leukaemic children receiving repeated cranial irradiation | **Scopus** |
|  | Factor analysis and validity of the conners parent and teacher rating scales in childhood cancer survivors | **Scopus** |
|  | Glutamine for the treatment of vincristine-induced neuropathy in children and adolescents with cancer | **Scopus** |
|  | Clinical Field Testing of an Enhanced-Activity Intervention in Hospitalized Children with Cancer | **Scopus** |
|  | **Decrease in motor performance in children with cancer is independent of the cumulative dose of vincristine** | **Scopus** |
|  | **Decrease in peripheral muscle strength and ankle dorsiflexion as long-term side effects of treatment for childhood cancer** | **Scopus** |
|  | Effects of 6 months ALL treatment on psychosocial development and emotional status in children aged 1 to 3 years [Altı Aylık ALL Tedavisinin 1-3 Yaşlarındaki Çocuklarda Psikososyal Gelişim ve Duygu Durumu Üzerindeki Etkileri] | **Scopus** |
|  | Neuropsychological outcome of children undergoing bone marrow transplantation | **Scopus** |
|  | Short-Term Recovery of Balance Control: Association with Chemotherapy-Induced Peripheral Neuropathy in Pediatric Oncology | **Scopus** |
|  | The measurement and diagnostic utility of intrasubtest scatter in pediatric neuropsychology | **Scopus** |
|  | Quinolinic acid levels in a murine retrovirus-induced immunodeficiency syndrome | **Scopus** |
|  | Successful outcome of allo-SCT in high-risk pediatric AML using chemotherapy-only conditioning and post transplant immunotherapy | **Scopus** |
|  | Nocturnal awakenings, sleep environment interruptions, and fatigue in hospitalized children with cancer | **Scopus** |
|  | Reintegration after bone marrow transplantation | **Scopus** |
|  | Cortical Morphometry and Its Relationship with Cognitive Functions in Children after non-CNS Cancer | **Scopus** |
|  | Impairments of Lower Extremity Muscle Strength and Balance in Childhood Cancer Patients and Survivors: A Systematic Review | **Scopus** |
|  | Safety of Symptom-Based Modification of Physical Therapy Interventions in Pediatric Oncology Patients with and Without Low Blood Counts | **Scopus** |
|  | Motor performance in children diagnosed with cancer: A longitudinal observational study | **Scopus** |
|  | The Effects of Exercise Prescription on Aerobic Performance and Quality of Life During the Course of Lymphoma Chemotherapy: Results of a Prospective Controlled Study | **Scopus** |
|  | Association of Neuronal Injury in the Genu and Body of Corpus Callosum After Cranial Irradiation in Children With Impaired Cognitive Control: A Prospective Study | **Scopus** |
|  | Fractures among long-term survivors of childhood cancer: A report from the Childhood Cancer Survivor Study | **Scopus** |
|  | Clinical uses of melatonin: Evaluation of human trials | **Scopus** |
|  | Neuropsychologic impairment in adult bone marrow transplant candidates | **Scopus** |
|  | Elevated visual dependency in young adults after chemotherapy in childhood | **Scopus** |
|  | Oculomotor deficits after chemotherapy in childhood | **Scopus** |
|  | Short-term recovery of chemotherapy-induced peripheral neuropathy after treatment for pediatric non-CNS cancer | **Scopus** |
|  | Social, emotional, and behavioral functioning of children with cancer | **Scopus** |
|  | ATSDR evaluation of health effects of benzene and relevance to public health | **Scopus** |
|  | Rehabilitation of therapy-related cognitive deficits in patients after hematopoietic stem cell transplantation | **Scopus** |
|  | Neurobehavioral toxicity of total body irradiation: A follow-up in long- term survivors | **Scopus** |
|  | Challenges Evaluating Chemotherapy-Induced Peripheral Neuropathy in Childhood Cancer Survivors: Which Instrument Should Nurses Use? | **Scopus** |
|  | Effects of Intraventricular Methotrexate on Neuronal Injury and Gene Expression in a Rat Model: Findings From an Exploratory Study | **Scopus** |
|  | Cognitive, behavioral, and social outcome in survivors of childhood stem cell transplantation | **Scopus** |
|  | Accuracy of perceived physical activity and fitness levels among childhood cancer survivors | **Scopus** |
|  | Graded aerobic exercise (GAEx): An effective exercise regimen to improve cardio-respiratory fitness and physical and psychosocial functioning in children with burn sequelae of the chest | **Scopus** |
|  | The Working Memory Network and Its Association with Working Memory Performance in Survivors of non-CNS Childhood Cancer | **Scopus** |
|  | Neuropsychological outcomes of children with Optic Pathway Glioma | **Scopus** |
|  | Physical rehabilitation practices for children and adolescents with cancer in Canada | **Scopus** |
|  | Loss of Adaptive Myelination Contributes to Methotrexate Chemotherapy-Related Cognitive Impairment | **Scopus** |
|  | A scoping review of physical therapy interventions for childhood cancers | **Scopus** |
|  | Prechemotherapy Levels of Plasma Dehydroepiandrosterone and Its Sulfated Form as Predictors of Cancer-Related Cognitive Impairment in Patients with Breast Cancer Receiving Chemotherapy | **Scopus** |
|  | Methotrexate Chemotherapy Induces Persistent Tri-glial Dysregulation that Underlies Chemotherapy-Related Cognitive Impairment | **Scopus** |
|  | Long-term psychological and educational outcomes for survivors of neuroblastoma: A report from the Childhood Cancer Survivor Study | **Scopus** |
|  | Developmental stage affects cognition in children with recently-diagnosed symptomatic focal epilepsy | **Scopus** |
|  | Subclinical pretreatment sensory deficits appear to predict the development of pain and numbness in patients with multiple myeloma undergoing chemotherapy | **Scopus** |
|  | Association between chemotherapy and cognitive impairments in a large cohort of patients with colorectal cancer | **Scopus** |
|  | Relationship between the number of life events and memory capacity in children | **Scopus** |
|  | Relationship between chemotherapy use and cognitive impairments in older women with breast cancer: Findings from a large population-based cohort | **Scopus** |
|  | Remediation of learning difficulties in children after treatment for a cerebellar medulloblastoma: A single-case study | **Scopus** |
|  | Neurocognitive function profile in HIV-infected school-age children | **Scopus** |
|  | Cognitive Outcome in Children with Myelomeningocele and Perinatal Hydrocephalus: A Longitudinal Perspective | **Scopus** |
|  | Cognitive functions of young adults who survived childhood cancer | **Scopus** |
|  | Motor ability, physical self-concept and health-related quality of life in pediatric cancer survivors | **Scopus** |
|  | Physical functioning and rehabilitation for the cancer survivor | **Scopus** |
|  | Neuropsychological resiliency after treatment for advanced stage neuroblastoma | **Scopus** |
|  | Cognitive and adaptive behavior 1 and 3 years following bone marrow transplantation | **Scopus** |
|  | Concordance between Self-reported Symptoms and Clinically Ascertained Peripheral Neuropathy among Childhood Cancer Survivors: The St. Jude Lifetime Cohort Study | **Scopus** |
|  | Executive functions and cerebellar development in children | **Scopus** |
|  | Late Complications after Bone Marrow Transplantation in Children and Adolescents | **Scopus** |
|  | The mediational role of executive functions for the relationship between motor ability and academic performance in pediatric cancer survivors | **Scopus** |
|  | Multidisciplinary Rehabilitation within Pediatric Cancer Care: A Holistic Approach | **Scopus** |
|  | Motor performance after treatment for pediatric bone tumors | **Scopus** |
|  | Effect of virtual reality on adolescent pain during burn wound care | **Scopus** |
|  | Alpe d'huzes cancer rehabilitation (a-care) research: Four randomized controlled exercise trials and economic evaluations in cancer patients and survivors | **Scopus** |
|  | The sequelae of cranial irradiation on human cognition | **Scopus** |
|  | Generalised cerebral atrophy following temporal lobectomy for intractable epilepsy associated with mesial temporal sclerosis | **Scopus** |
|  | Current status of psychological research in organ transplantation | **Scopus** |
|  | Long-Term Neurobehavioral Outcome in Pediatric Brain-Tumor Patients: Review and Methodological Critique | **Scopus** |
|  | Cyclophosphamide impairs hippocampus-dependent learning and memory in adult mice: Possible involvement of hippocampal neurogenesis in chemotherapy-induced memory deficits | **Scopus** |
|  | Health-related quality of life in AYA cancer survivors who underwent HSCT compared with healthy peers | **Scopus** |
|  | The Brainfit study: Efficacy of cognitive training and exergaming in pediatric cancer survivors - a randomized controlled trial | **Scopus** |
|  | Physiological mechanisms, behavioral and psychological factors influencing the transfer of milk from mothers to their young | **Scopus** |
|  | Neuropeptide changes and neuroactive amino acids in CSF from humans and sheep with neuronal ceroid lipofuscinoses (NCLs, Batten disease) | **Scopus** |
|  | Relations between fatigue, neuropsychological functioning, and physical activity after treatment for breast carcinoma: Daily self-report and objective behavior | **Scopus** |
|  | Late cognitive and radiographic changes related to radiotherapy: Initial prospective findings | **Scopus** |
|  | Body image and psychosocial adjustment in adolescent cancer survivors | **Scopus** |
|  | Medical and Pharmacologic Treatment of Learning Disabilities | **Scopus** |
|  | Psychiatric and Psychosocial Aspects of Bone Marrow Transplantation | **Scopus** |
|  | Cognitive function of patients with nasopharyngeal carcinoma with and without temporal lobe radionecrosis | **Scopus** |
|  | Validation of a haptic-based simulation to test complex figure reproduction capability | **Scopus** |
|  | Memory performance used to detect radiation effects on cognitive functioning | **Scopus** |
|  | The change in fatigue, strength, and quality of life following a physical therapist prescribed exercise program for cancer survivors | **Scopus** |
|  | Radiotherapeutic effects on brain function: Double dissociation of memory systems | **Scopus** |
|  | Does frontal normality exist in schizophrenia? A saccadic eye movement study | **Scopus** |
|  | Adaptation to an autoimmune disorder: Does mental flexibility impact illness-related self-regulation? | **Scopus** |
|  | Milk osteopontin promotes brain development by up-regulating osteopontin in the brain in early life | **Scopus** |
|  | Fenretinide Beneficial Effects on Amyotrophic Lateral Sclerosis-associated SOD1G93A Mutant Protein Toxicity: In Vitro and In Vivo Evidences | **Scopus** |
|  | Hand-held dynamometry in patients with haematological malignancies: Measurement error in the clinical assessment of knee extension strength | **Scopus** |
|  | The use of the Godin-Shephard Leisure-Time Physical Activity Questionnaire in oncology research: a systematic review | **Scopus** |
|  | The effects of exercise interventions on quality of life in clinical and healthy populations; a meta-analysis | **Scopus** |
|  | Transcriptomic profiling of skeletal muscle from the ts1cje mouse model of down syndrome suggests dysregulation of trisomic genes associated with neuromuscular junction signaling, oxidative stress and chronic inflammation | **Scopus** |
|  | The role of svz stem cells in glioblastoma | **Scopus** |
|  | Lysine acetyltransferases CBP and p300 as therapeutic targets in cognitive and neurodegenerative disorders | **Scopus** |
|  | Hormonal regulation of oligodendrogenesis i: Effects across the lifespan | **Scopus** |
|  | Static fields | **Scopus** |
|  | HIV-1 and drug abuse comorbidity: Lessons learned from the animal models of NeuroHIV | **Scopus** |
|  | Thermology 2012 - A computer-assisted literature survey | **Scopus** |
|  | Handwriting and fine motor problems after treatment for acute lymphoblastic leukemia | **WOS** |
|  | Motor functioning during and following treatment with chemotherapy for pediatric acute lymphoblastic leukemia | **WOS** |
|  | Fine Motor Skills in Pre-School Children with Acute Lymphoblastic Leukemia Compared with Healthy Peers: A Preliminary Study | **WOS** |
|  | Assessment of late sequelae after CNS relapse of childhood acute lymphoblastic leukemia | **WOS** |
|  | Mathematics Development and Difficulties: The Role of Visual-Spatial Perception and Other Cognitive Skills | **WOS** |
|  | Gross motor disorders in pediatric patients with acute lymphoblastic leukemia and survivors: A systematic review. | **WOS** |
|  | SPEECH AND LANGUAGE PERFORMANCE OF BRAIN-INJURED CHILDREN | **WOS** |
|  | Assessment of Gross Motor Skills and Phenotype Profile in Children 9-11 Years of Age in Survivors of Acute Lymphoblastic Leukemia | **WOS** |
|  | Chemotherapeutic CNS prophylaxis and neuropsychologic change in children with acute lymphoblastic leukemia: A prospective study | **WOS** |
|  | Low Cerebellar Vermis Volumes and Impaired Neuropsychologic Performance in Children Treated for Brain Tumors and Leukemia | **WOS** |
|  | Proficiency of balance in children and youth who have had acute lymphoblastic leukemia | **WOS** |
|  | Tc-99m-HMPAO brain perfusion single-photon emission computed tomography in children with Down syndrome: Relationship to epilepsy, thyroid functions, and congenital heart disease | **WOS** |
|  | Math weaknesses in survivors of acute lymphoblastic leukemia compared to healthy children | **WOS** |
|  | Neuropsychological outcome following intensity-modulated radiation therapy for pediatric medulloblastoma | **WOS** |
|  | HYPOPLASIA OF THE CEREBELLAR VERMIS AND COGNITIVE DEFICITS IN SURVIVORS OF CHILDHOOD LEUKEMIA | **WOS** |
|  | Modifying bone mineral density, physical function, and quality of life in children with acute lymphoblastic leukemia | **WOS** |
|  | Strength and functional mobility in children with acute lymphoblastic leukemia | **WOS** |
|  | The contribution of neurocognitive functioning to quality of life after childhood acute lymphoblastic leukemia | **WOS** |
|  | CRANIAL COMPUTED-TOMOGRAPHY OF 64 CHILDREN IN CONTINUOUS COMPLETE REMISSION OF LEUKEMIA .2. RELATIONS TO PATIENT DATA AND NEUROLOGICAL COMPLICATIONS | **WOS** |
|  | Long-term neuropsychological risks in pediatric bone marrow transplant: What do we know? | **WOS** |
|  | NEUROPSYCHOLOGICAL SEQUELAE OF CHILDHOOD-CANCER IN LONG-TERM SURVIVORS | **WOS** |
|  | The ecological validity of the Rey-Osterrieth Complex Figure: Predicting everyday problems in children with neuropsychological disorders | **WOS** |
|  | Outcome of the Rotterdam-84 CNS-ALL chemotherapy protocol without radiotherapy for isolated central nervous system relapsed acute lymphoblastic leukemia. | **WOS** |
|  | Association between radiation dose to neuronal progenitor cell niches and temporal lobes and performance on neuropsychological testing in children: a prospective study | **WOS** |
|  | The effect of game-based exercise on infant acute lymphocytic leukaemia patients | **WOS** |
|  | Decreased postural control in adult survivors of childhood cancer treated with chemotherapy | **WOS** |
|  | Assessment of regional GABA(A) receptor binding using F-18-fluoroflumazenil positron emission tomography in spastic type cerebral palsy | **WOS** |
|  | Late effects of central nervous system prophylactic leukemia therapy on cognitive functioning. | **WOS** |
|  | Impairment of expressive behavior in pediatric HIV-infected patients with evidence of CNS disease | **WOS** |
|  | UNUSUAL PALM CREASES AND UNUSUAL CHILDREN - SYDNEY LINE AND TYPE-C PALMAR LINES AND THEIR CLINICAL SIGNIFICANCE IN A CHILD DEVELOPMENT CLINIC | **WOS** |
|  | NEUROBEHAVIORAL AND PSYCHOSOCIAL FUNCTIONING OF CHILDREN WITH OPSOCLONUS-MYOCLONUS SYNDROME | **WOS** |
|  | Visual perceptual skills and visual motor integration in children and adolescents after allogeneic hematopoietic stem cell transplantation | **WOS** |
|  | Neuropsychological effects of prophylactic cranial irradiation (PCI) in adult cancer patients | **WOS** |
|  | Ataxia-telangiectasia in Iran: Clinical and laboratory features of 104 patients | **WOS** |
|  | Late sequelae of CNS recurrence of acute lymphoblastic leukemia in childhood | **Pub M** |
|  | Improving Occupational Performance in Pediatric Hematopoietic Cell Transplant Recipients. | **Pub M** |
|  | Motor and cognitive testing of bone marrow transplant patients after chemoradiotherapy. | **Pub M** |
|  | Intellectual outcome in children and adolescents with acute lymphoblastic leukaemia treated with chemotherapy alone: age- and sex-related differences | **SD** |
|  | **Slight cognitive impairment and magnetic resonance imaging abnormalities but normal school levels in children treated for acute lymphoblastic leukemia with chemotherapy only** | **SD** |
|  | Measurement of balance in survivors of acute lymphoblastic leukemia in childhood | **SD** |
|  | Allogeneic hematopoietic cell transplantation for infants with acute lymphoblastic leukemia | **SD** |
|  | Neuropsychological, Neuroanatomical, and Neurophysiological Consequences of CNS Chemotherapy for Acute Lymphoblastic Leukemia | **SD** |
|  | Neuromotor and neuropsychological manifestations of “total therapy” in children with acute lymphoblastic leukemia | **SD** |
|  | Efficacy and Morbidity of Central Nervous System “Prophylaxis” in Childhood Acute Lymphoblastic Leukemia: Eight Years Experience With Cranial Irradiation and Intrathecal Methotrexate | **SD** |
|  | Vincristine-induced vocal cord paresis and paralysis in children | **SD** |
|  | Intellectual functioning of childhood leukemia survivors - relation to Tau protein - a marker of white matter injury | **SD** |
|  | Late effects among survivors of leukemia during childhood and adolescence | **SD** |
|  | Symptom Clusters in Children With Cancer | **SD** |
|  | Survival and Neurocognitive Outcomes After Cranial or Craniospinal Irradiation Plus Total-Body Irradiation Before Stem Cell Transplantation in Pediatric Leukemia Patients With Central Nervous System Involvement | **SD** |
|  | Safety and Feasibility of Rehabilitation Interventions in Children Undergoing Hematopoietic Stem Cell Transplant WithThrombocytopenia | **SD** |
|  | Primary Care of Children and Adolescents with Down Syndrome: An Update | **SD** |
|  | Neurocognitive Effects and Necrosis in Childhood Cancer Survivors Treated With Radiation Therapy: A PENTEC Comprehensive Review | **SD** |
|  | Cognitive status of children treated with central nervous system prophylactic chemotherapy for acute lymphocytic leukemia | **SD** |
|  | Psychological studies of children who have cancer | **SD** |
|  | Effect of continuous-infusion zidovudine therapy on neuropsychologic functioning in children with symptomatic human immunodeficiency virus infection | **SD** |
|  | Cognitive and psychosocial sequelae for children with acute lymphocytic leukemia and their families | **SD** |
|  | The Arthropathy of Down Syndrome: An Underdiagnosed and Under-recognized Condition | **SD** |
|  | Duration of chronic kidney disease reduces attention and executive function in pediatric patients | **SD** |
|  | Multiple giant cell lesions in a patient with Noonan syndrome with multiple lentigines | **SD** |
|  | Characterization of a novel variant in siblings with Asparagine Synthetase Deficiency | **SD** |
|  | Pediatric Brain Tumors | **SD** |
|  | Related visual impairment to mother-infant interaction and development in infants with bilateral retinoblastoma | **SD** |
|  | A Clinical Study of Sotos Syndrome Patients With Review of the Literature | **SD** |
|  | Chronobiology and chronotherapy in medicine | **SD** |
|  | Neurobehavioral Manifestations and Sequelae of HIV and Other Infections | **SD** |
|  | Physical Activity in Pediatric Cancer patients with solid tumors (PAPEC): Trial rationale and design | **SD** |
|  | ONCOLOGIC EMERGENCIES | **SD** |
|  | Childhood cancer: Sequelae — the cost of cure | **SD** |
|  | Chemobrain: A translational challenge for neurotoxicology | **SD** |
|  | Executive Function Following Focal Frontal Lobe Lesions: Impact of Timing of Lesion on Outcome | **SD** |
|  | Long-Term Cognitive Sequelae After Pediatric Brain Tumor Related to Medical Risk Factors, Age, and Sex | **SD** |
|  | Late effects of treatment in long-term survivors of cancer | **SD** |
|  | Survivorship in young patients with bone cancer | **SD** |
|  | Noonan syndrome: genetic and clinical update and treatment options | **SD** |
|  | Graphology | **SD** |
|  | Organisation of follow-up in paediatric oncology | **SD** |
|  | Neurocognitive late effects in pediatric cancer | **SD** |
|  | Persistent, bioaccumulative and toxic substances in fish: Human health considerations | **SD** |
|  | Rituximab therapy for multisystem autoimmune diseases in pediatric patients | **SD** |
|  | Altered consciousness and shock in a malnourished child | **SD** |
|  | Leisure-time physical activity and psychosocial well-being in adolescents after cancer diagnosis | **SD** |
|  | A 7-Year-Old Girl with Cerebral Palsy and Multiple Warts | **SD** |
|  | Potential role of proton therapy in the treatment of pediatric medulloblastoma/primitive neuroectodermal tumors: Reduction of the supratentorial target volume | **SD** |
|  | Medical perspectives on pediatric sports medicine–Selective topics | **SD** |
|  | Folinic acid therapy in treatment of dihydropteridine reductase deficiency | **SD** |
|  | Biological effects after prenatal irradiation (embryo and fetus): ICRP Publication 90 Approved by the Commission in October 2002 | **SD** |
|  | Constitutional chromosome aberrations as pathogenetic events in hematologic malignancies | **SD** |
|  | Bone marrow transplantation for genetic disorders | **SD** |
|  | New Drugs of 2001 | **SD** |
